# Effects of induction and inhibition of matrix cross-linking on remodeling of the aqueous outflow resistance by ocular trabecular meshwork cells

**DOI:** 10.1038/srep30505

**Published:** 2016-07-28

**Authors:** Yong-Feng Yang, Ying Ying Sun, Ted S. Acott, Kate E. Keller

**Affiliations:** 1 Casey Eye Institute, Oregon Health & Science University, 3181 Sam Jackson Park Road, Portland, OR 97219, USA

## Abstract

The trabecular meshwork (TM) tissue controls drainage of aqueous humor from the anterior chamber of the eye primarily by regulating extracellular matrix (ECM) remodeling by matrix metalloproteinases (MMPs). Glaucomatous TM tissue is stiffer than age-matched controls, which may be due to alterations in ECM cross-linking. In this study, we used genipin or beta-aminopropionitrile (BAPN) agents to induce or inhibit matrix cross-linking, respectively, to investigate the effects on outflow resistance and ECM remodeling. Treatment with BAPN increased outflow rates in perfused human and porcine anterior segments, whereas genipin reduced outflow. Using a fluorogenic peptide assay, MMP activity was increased with BAPN treatment, but reduced with genipin treatment. In genipin-treated TM cells, Western immunoblotting showed a reduction of active MMP2 and MMP14 species and the presence of TIMP2-MMP14 higher molecular weight complexes. BAPN treatment increased collagen type I mRNA and protein levels, but genipin reduced the levels of collagen type I, tenascin C, elastin and versican. CD44 and fibronectin levels were unaffected by either treatment. Collectively, our results show that matrix cross-linking has profound effects on outflow resistance and ECM composition and are consistent with the emerging paradigm that the stiffer the ECM, the lower the aqueous outflow facility through the TM.

The trabecular meshwork (TM) regulates drainage of aqueous humor from the anterior chamber to Schlemm’s canal^1^. Resistance to aqueous humor outflow is generated in order to establish uniform intraocular pressure (IOP). The probable site of the outflow resistance is located within the deepest portion of the TM, in a region called the juxtacanalicular region (JCT) and the inner wall basement membrane of Schlemm’s canal^1^,^2^,^3^,^4^,^5^. Outflow resistance is thought to be comprised primarily of extracellular matrix (ECM) with some important contribution from the actin cytoskeleton of TM cells and SC inner wall cells^2^,^6^,^7^. Matrix metalloproteinase (MMP) proteolytic activity is required to maintain outflow facility^8^,^9^,^10^,^11^,^12^. Increased perfusion pressure in anterior segment organ culture increases MMP activity, which degrades existing ECM and triggers numerous changes in ECM gene expression levels^11^,^13^. Replacement ECM is slightly different in composition and/or organization in order to maintain the modified outflow resistance. Thus, a tunable ECM system is established that can respond to sustained pressure increases in order to reduce IOP.

MMPs -2 and -14 are essential to remodeling of the JCT and are constitutively expressed at relatively high levels^10^,^11^,^14^. Both enzymes are synthesized as inactive pro-forms. MMP2 activation relies on formation of a complex with MMP14 and tissue inhibitor of MMP-2 (TIMP2)^15^,^16^,^17^. In the complex, the N-terminus of TIMP2 binds the catalytic site of one MMP14 molecule, which then acts as a receptor for proMMP2. An adjacent TIMP2-free MMP14 molecule then cleaves the pro-peptide from proMMP2 and further autocatalytic processing generates fully active MMP2. The concentration of TIMP2 dictates the activity level of MMP2: when TIMP2 levels increase, more ternary complexes of MMP14-TIMP2-MMP2 are formed and MMP2 activity is increased^18^. However, a tipping point is reached where very high levels of TIMP2 actually inhibit activation of MMP2 because TIMP2 directly binds to MMP2 thereby sequestering it from the activation complex^18^. Thus, the balance between the levels of these three molecules can either promote or inhibit MMP2 activation.

The ECM provides structural and mechanical support for cells in tissues^19^. The major structural macromolecules are collagen fibrils, which impart tensile strength to the tissue, and elastin microfibrils, which confer elasticity and allows tissues to adapt to repetitive mechanical stresses^19^. Modifications such as enzymatic cross-linking of collagen and elastin fibers provide increased structural integrity and durability to a tissue^20^. A major class of enzymes that is involved in collagen cross-linking is the lysyl oxidases (LOX)^20^,^21^. These are a family of five copper-dependent monoamine oxidases that modify ε-amino groups of lysine residues in collagen and elastin precursors to allysine, an aldehyde product^22^. These aldehydes are highly reactive and cross-links spontaneously occur between other aldehydes or between unmodified lysine residues. Activation of LOX stabilizes collagen and elastin fibrils making them insoluble and resistant to proteolytic degradation. Conversely, reducing LOX activity reduces tissue stiffness^23^.

As tissue age, they become mechanically weaker, less elastic and more rigid than younger tissue^24^,^25^,^26^. Several studies have linked alterations in tissue stiffness to glaucoma. In the TM, atomic force microscopy was used to show that TM tissue from primary open-angle glaucoma (POAG) patients was significantly stiffer than age-matched control TM^27^. Moreover, Schlemm’s canal cells from glaucoma patients were stiffer than normal control SC cells^28^. Pore formation in glaucomatous SC cells was significantly reduced, which could contribute to the increased resistance to aqueous humor outflow in glaucoma patients. Furthermore, in 2007, a genome-wide association study identified two single nucleotide polymorphisms (SNPs) in LOX-like-1 (*LOXL1*) that were significantly associated with exfoliation syndrome, a common cause of open-angle glaucoma^29^. However, the relationship between LOX cross-linking, tissue rigidity and IOP remains unclear.

Cross-linking can be induced and inhibited using chemicals called genipin and beta-aminopropionitrile (BAPN), respectively. Genipin is a natural collagen cross-linking reagent derived from the Gardenia fruit, which has been widely used in traditional Chinese medicine^30^. It reacts with free amino groups on lysine, hydroxylysine or arginine residues and forms intra-molecular and intermolecular crosslinks with a cyclic structure within collagen fibers in biological tissue^31^,^32^. The lathyrogen, BAPN, is derived from the peas of *Lathyrus* plants. BAPN irreversibly inhibits the conversion of lysine residues to aldehydes by LOX in collagens and elastin^22^. In this study, we investigated the effects of inducing (genipin) and inhibiting (BAPN) matrix cross-linking on outflow rates in anterior segment perfusion culture and investigated the effects of these cross-linking agents on MMPs and other ECM molecules that are thought to control or comprise the outflow resistance.

## Results

**Figure 1:**
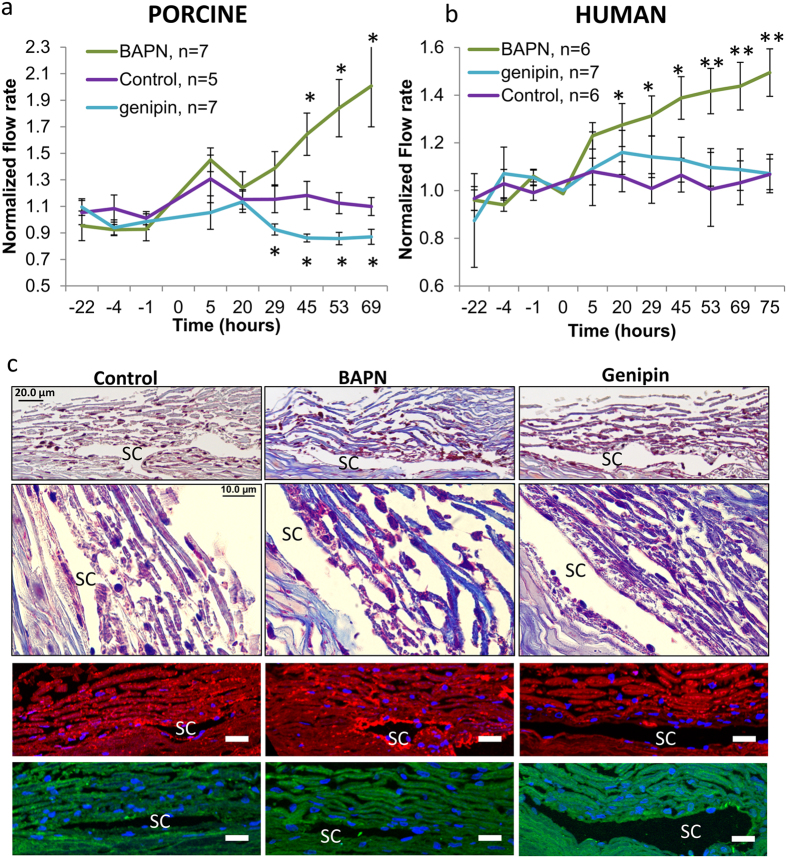
The effects of matrix cross-linking agents on outflow rates in perfusion culture. BAPN (cross-link inhibitor) or genipin (cross-link inducer) were added at time point “0” to (**a**) porcine and (**b**) human anterior segments in perfusion culture. Outflow rates were monitored for a further 69-75 hours. Average outflow facilities at 75 hours after treatment were 0.44 (control), 0.494 (BAPN) and 0.159 (genipin) μl/min/mm Hg for porcine anterior segments and 0.365 (control), 0.489 (BAPN) and 0.321 (genipin) μl/min/mm Hg for human anterior segments. Error bars are the standard error of the mean. *p < 0.05; **p < 0.01. (**c**) Masson’s trichrome histological staining of human TM tissue post-perfusion at 20× and 100×. Immunostaining with fibronectin (red) and fibrillin-1 antibodies (green) are also shown. The cornea is oriented toward the left of each image and the sclera at the right. SC = Schlemm’s canal. Scale bars for immunostained images = 20 μm.

To assess the effects of cross-linking on outflow resistance, human and porcine anterior segments were perfused with BAPN or genipin (Fig. 1). BAPN was found to significantly increase outflow rates approximately 1.5- to 2-fold in porcine (Fig. 1a) and human (Fig. 1b) perfusion cultures. Conversely, genipin decreased flow rates to approximately 0.85 in porcine anterior segments, but had no significant effect on flow rates of perfused human eyes compared to vehicle control. Average outflow facilities (C = flow rate / pressure) at the final time point were 0.44 (control), 0.494 (BAPN) and 0.159 (genipin) μl/min/mm Hg for porcine anterior segments and 0.365 (control), 0.489 (BAPN) and 0.321 (genipin) μl/min/mm Hg for human anterior segments.

Masson’s trichrome staining of human tissue after perfusion (Fig. 1c) showed increased collagen staining (blue) in BAPN-treated TM compared to control. Nuclei (black) were present in all sections showing that treatments were not toxic to TM cells and that the outflow effects were not due to cell loss. Immunostaining with fibronectin (red) antibodies showed an apparent increase in fibronectin immunostaining of the JCT and inner wall in BAPN-treated TM, whereas there was reduced immunostaining in these regions in genipin-treated TM. However, fibrillin-1 (green) antibodies showed few differences in distribution between treatments.

**Figure 2:**
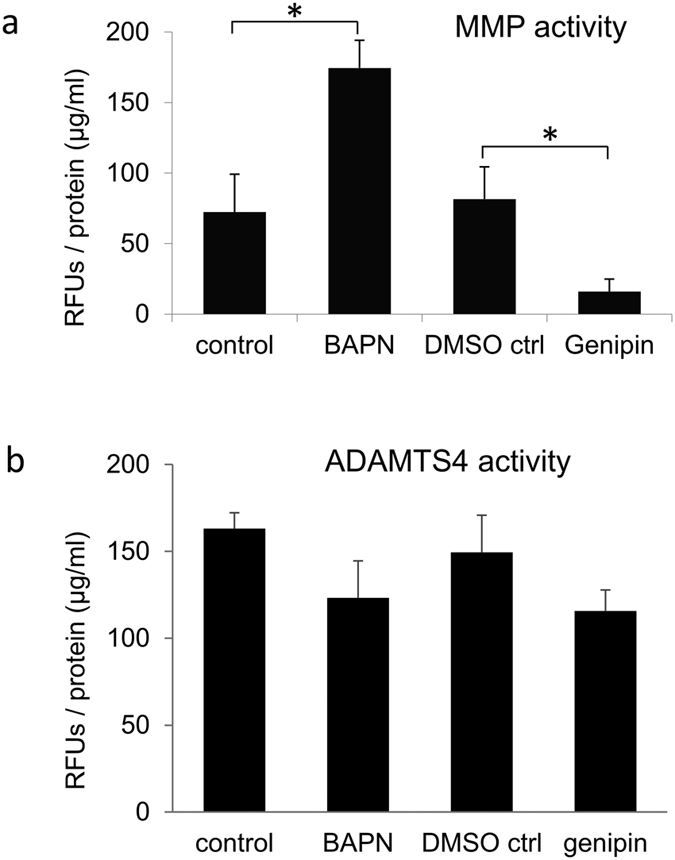
MMP and ADAMTS4 activity in TM cells treated with BAPN or genipin. (**a**) MMP activity in the media of TM cells treated with BAPN and genipin. Results show the mean RFUs ± standard error following normalization to total protein in each sample. N = 3; *p < 0.05. (**b**) ADAMTS4 activity in RIPA lysates of TM cells treated with BAPN and genipin. Results show the mean RFUs ± standard error following normalization to total protein in each sample. N = 3.

Modulating levels and activities of MMPs is the principal mechanism by which TM cells remodel their ECM to alter aqueous outflow resistance^11^,^12^,^33^. Therefore, we investigated the effects of the cross-linking agents on MMP activity (Fig. 2a). Using a fluorogenic assay, we found that BAPN significantly induced MMP activity approximately 2.5-fold, while genipin was a potent inhibitor of MMP activity, which was approximately 5-fold less than vehicle controls (Fig. 2a). We also investigated ADAMTS4 activity since this is an enzyme that cleaves versican and increases outflow in perfusion culture^34^. ADAMTS4 activity was not significantly different with BAPN or genipin treatment in cell lysates compared to control cell cultures (Fig. 2b).

**Figure 3:**
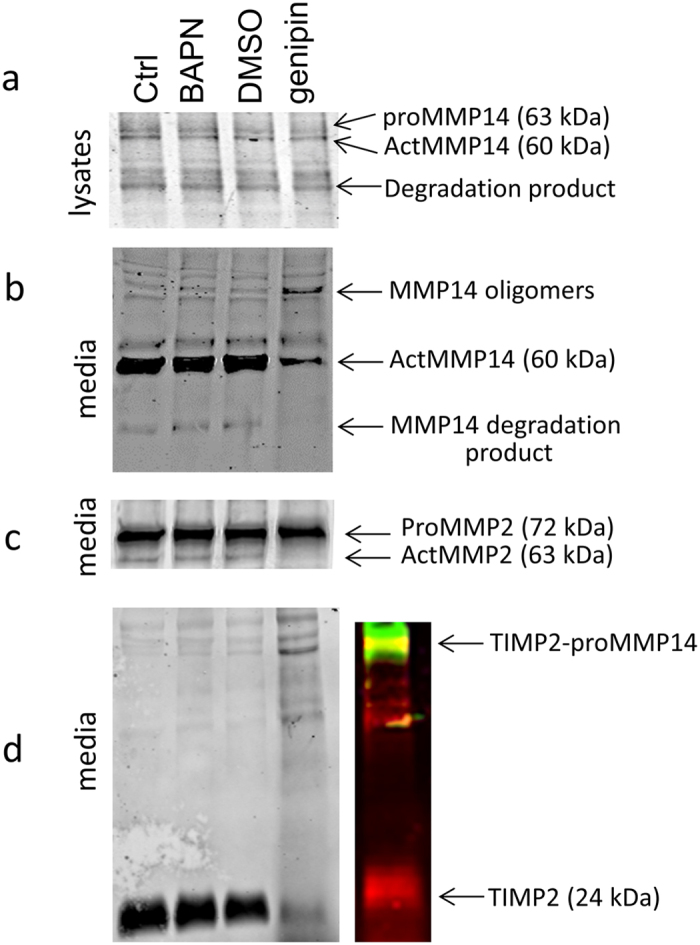
Effects of BAPN and genipin on MMP2, MMP14 and TIMP2. Western immunoblots of (**a**,**b**) MMP14, (**c**) MMP2 and (**d**) TIMP2 of control, BAPN and genipin-treated TM cells in culture for 48 hours. Two controls are included: serum-free media control (Ctrl) for BAPN and DMSO as the vehicle control for genipin. The expected sizes of the pro- and active forms of the enzymes are indicated. The colored panel shows both TIMP2 (red) and MMP14 (green) antibodies on the same immunoblot. Yellow shows the bands that are detected by both antibodies.

To investigate whether the differences in MMP activity were due to alterations in protein levels, Western immunoblotting with antibodies against MMP2 and MMP14 was performed (Fig. 3). No significant differences in MMP protein levels were observed for BAPN-treated TM cells compared to control. Conversely, the results show that the active forms of MMP2 (63 kDa) and MMP14 (60 kDa) were highly reduced in the media of genipin-treated TM cells. The precursor form of MMP2 (72 kDa) was not substantially affected by genipin treatment. Interestingly, for MMP14, there appeared to be accumulation of a higher molecular weight product (approx. 160 kDa) in the media (Fig. 3b). This may represent MMP14 oligomers or MMP14-TIMP2 complexes, which have been previously reported^15^,^35^. To investigate this possibility, Western immunoblotting with TIMP2 antibodies was performed (Fig. 3d). In the media, TIMP2 was detected at 24 kDa in control and BAPN-treated TM cells as expected, with smaller amounts of higher molecular weight species. However, in genipin-treated TM cells, the higher molecular weight complexes were enriched. When MMP14 (green) and TIMP2 (red) antibodies were used on the same gel, some of these bands apparently co-migrated (yellow). These are likely to represent the TIMP2-MMP14 complex and its dimer.

**Figure 4:**
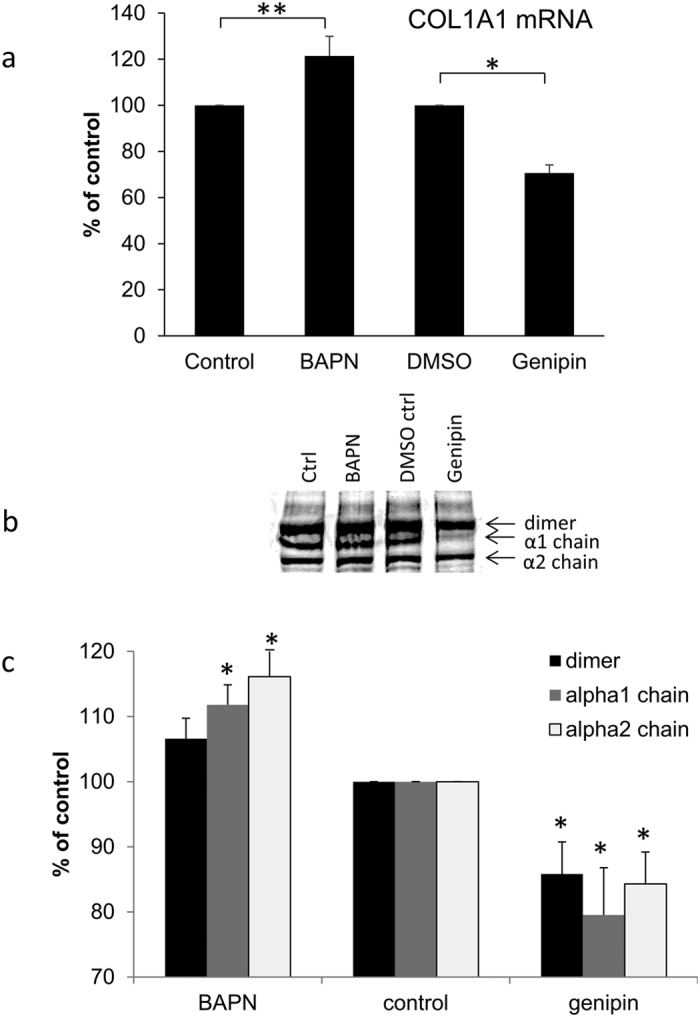
Effects of BAPN and genipin on collagen type I. (**a**) Quantitative RT-PCR of *COL1A1* mRNA in TM cells treated for 24 hours with BAPN or genipin. Results are shown as a percentage of their respective controls. Data are average ± standard error of the mean. N = 4; *p = 0.001; **p = 0.021. (**b**) Representative Western immunoblots of collagen type I synthesized by TM cells treated for 48 hours with BAPN or genipin. The positions of the α1 and α2 collagen chains and their dimer are shown. Molecular weights are shown in kDa. (**c**) Densitometry of each of the three bands relative to its control. Data are average ± standard error of the mean. N = 3; *p < 0.05.

Next, we investigated the effects of BAPN and genipin on the protein levels of other ECM molecules. First, we investigated collagen type I (Fig. 4). Quantitative RT-PCR showed a significant increase in COL1A1 mRNA after 24 hours treatment with BAPN and conversely, a decrease with genipin treatment (Fig. 4a). Western immunoblotting and densitometry also showed that collagen α1 and α2 chains were significantly increased in the media after 48 hours with BAPN treatment, but collagen I was reduced with genipin treatment (Fig. 4b,c).

**Figure 5:**
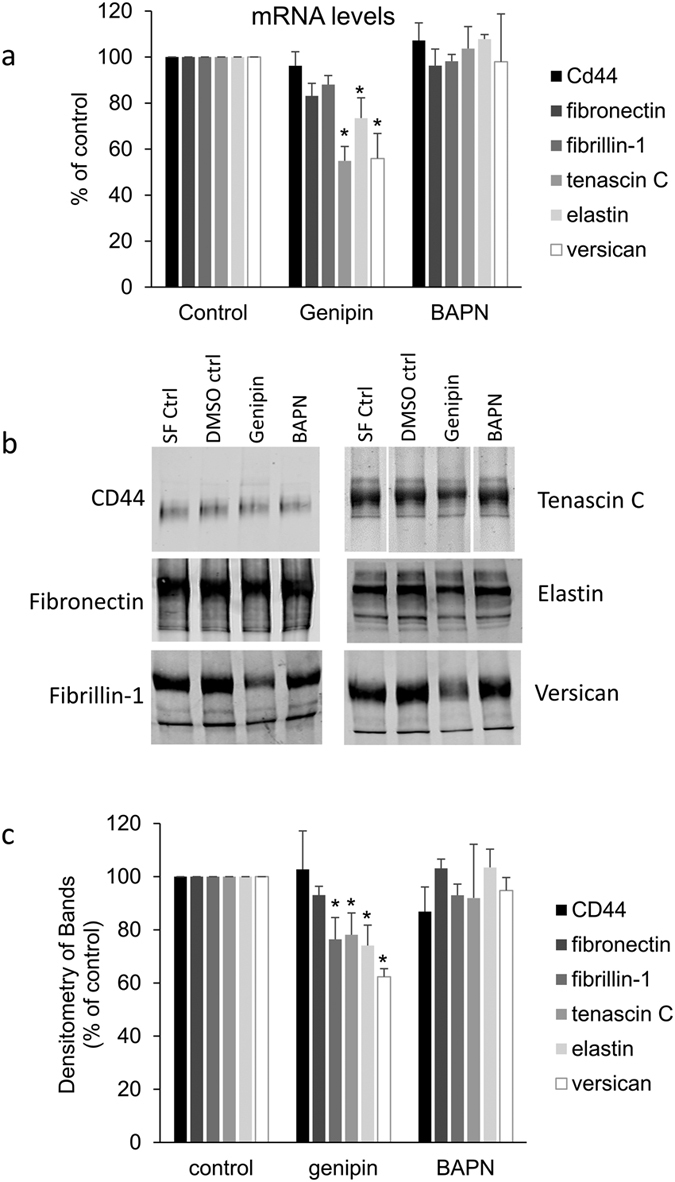
Effects of BAPN and genipin on other ECM molecules. (**a**) Quantitative RT-PCR of mRNA for various ECM molecules in TM cells treated for 24 hours with BAPN or genipin. Results are shown as a percentage of their respective controls. Data are average ± standard error of the mean. N = 4–6; *p < 0.05. (**b**) Representative Western immunoblots for CD44, fibronectin, fibrillin-1, tenascin C, elastin and versican produced by TM cells treated for 48 hours with BAPN or genipin. Serum-free (SF) control is the vehicle for BAPN and DMSO is the control for genipin. (**c**) Densitometry of each of the bands relative to its control. When multiple bands were present, the strongest band was evaluated. Data are average ± standard error of the mean. N = 3; *p < 0.05.

Finally, we investigated ECM molecules that either influence aqueous outflow resistance (versican, CD44, fibronectin), or are components of elastic fibers (fibrillin-1, elastin) and other proteins that organize ECM structure (tenascin C). By quantitative RT-PCR, tenascin C, elastin and versican mRNAs were significantly decreased by genipin treatment after 24 hours, but BAPN did not appear to have a significant effect (Fig. 5). Western immunoblotting and densitometry confirmed that tenascin C, elastin and versican protein levels in the media were reduced at 48 hours by genipin treatment and fibrillin-1 protein was also significantly reduced (Fig. 5b,c). However, fibronectin and CD44 levels were not significantly affected by either cross-linking agent.

## Discussion

In this study, we investigated the effects of two agents that induce or inhibit matrix cross-linking on aqueous outflow resistance. As expected, the results show that these agents have opposite effects on outflow: inhibition of cross-linking with BAPN increased outflow, while inducing cross-linking using genipin reduced outflow in porcine eyes. While BAPN increased outflow rates in both species, genipin did not have a significant effect on outflow in human anterior segments. One possible explanation for this is that the aged human tissue (average age = 79 years) was already highly cross-linked compared to young (<2 years) porcine TM and further induction of cross-linking by genipin had no additive effect. However, further studies are required to test whether eyes from younger human donors respond to genipin treatment. Nevertheless, our outflow results are consistent with use of these cross-linking agents in animal studies. In cynomolgus monkeys, who had received laser-induced glaucoma, BAPN was applied topically on the inferior fornix and as a daily intramuscular injection^36^. In this monkey model, there was a significant reduction in IOP compared to surgical treatment alone. Conversely, when genipin cross-linked chitosan was implanted into the anterior segments of rabbits, IOP was found to be significantly increased compared to a sham-operated group^37^. In another study, Young’s modulus values were dramatically increased in genipin-treated porcine sclera^38^. Collectively, these studies suggest a direct relationship between the level and nature of matrix cross-links, tissue stiffness and IOP.

Post-perfusion, there was apparent increase and decrease in fibronectin immunostaining of the JCT and inner wall in BAPN- and genipin-treated TM, respectively. However, many ECM proteins show segmental expression i.e. they display different expression levels in high and low outflow regions of the tissue^39^. Since fluorescent tracers were not used in this study to monitor outflow patterns, apparent changes in fibronectin in treated eyes may merely reflect different expression levels in high and low outflow regions.

The levels and activity of MMPs are crucial to ECM turnover and IOP homeostasis^10^,^11^. We therefore tested the effects of the cross-linking agents on MMPs in TM cells and found that BAPN treatment significantly increased MMP activity. Furthermore, collagen I levels were increased with BAPN treatment by Western immunoblotting, which is consistent with the increased blue staining in Masson-trichrome stained sections post-perfusion. While fibrillar collagens provide structural support for the tissue, they are unlikely to be a main source of outflow resistance. However, the collagen fibrillar network provides a platform that directs the assembly of other ECM components such as proteoglycans into a configuration to facilitate outflow. Together, our results suggest that induction of MMP activity and/or changes to the structural organization of outflow components by altered collagen type I levels are the most likely reasons for the observed increased outflow by BAPN treatment in perfusion culture. BAPN did not appear to alter mRNA or protein levels of any of the other ECM molecules tested. This is different to a prior study which showed increased elastin protein with BAPN treatment^40^. However, their study used a much higher BAPN concentration (>1 mM) than in our study.

In contrast to BAPN, genipin treatment significantly decreased MMP activity. Western immunoblotting also revealed a major difference in the MMP bands detected. Bands equivalent to the active enzymes of MMP14 (60 kDa) and MMP2 (63 kDa) were highly diminished in genipin lanes compared to DMSO vehicle control. Moreover, with MMP14 there was an apparent accumulation of a higher molecular weight species (~160 kDa). This band was also detected with the TIMP2 antibodies. We speculate that genipin cross-links the MMP14-TIMP2 into an inactive complex, which is unable to process MMP2 into its active form. Since MMP2 is one of the major MMPs that influences outflow^11^,^12^, these results may explain why outflow is reduced in genipin-treated anterior segments. However, genipin also reduced protein levels of collagen type I, fibrillin-1, tenascin C, elastin and versican. Thus, in addition to its effects on MMP levels and activity, genipin treatment reduces synthesis of molecules that affects both the collagenous network where ECM components are embedded, as well as several key components of the outflow resistance. However, only a small subset of ECM molecules was studied and it is likely that other ECM proteins may increase synthesis in response to genipin treatment. From this study and our prior assertions^1^,^12^,^39^,^41^, it is our contention is that outflow resistance is governed by the relative composition of ECM molecules in the outflow channels. The total amount of ECM remains relatively unaffected so that it would support the structural integrity of the tissue while targeted degradation of the resistance components by MMPs in the outflow channels adjusts outflow facility.

MMP14 is a transmembrane MMP, but somewhat surprisingly, MMP14 was found in the media of HTM cells. In other cell types, MMP14 is a component of exosomes^42^,^43^,^44^. These small vesicles are formed by inward budding of the plasma membrane, and following endosomal sorting into multi-vesicular bodies, they are recycled back to the cell surface and secreted^45^. Exosomes are released by TM cells in culture and are present in aqueous humor^46^,^47^. Exosomes derived from HT-1080 fibrosarcoma and G361 melanoma cells contain the catalytically active 60 kDa form of MMP14 as well as the proteolytically processed 43 kDa form^43^. In exosomes derived from corneal fibroblasts, MMP14 was present as a monomer as well as higher molecular weight oligomers and lower molecular weight degradation products, a pattern similar to MMP14 detected in the media of TM cells^44^. We used anti-TIMP2 to investigate whether these products could represent MMP14-TIMP2 complexes. We found that one of the bands detected is most likely the previously described 80 kDa band, which is comprised of TIMP2 bound to active MMP14, and its dimer (~160 kDa)^48^. Interestingly, TIMP2 was reported to be a component of exosomes isolated from a HTM cell culture by mass spectrometry^49^. Thus, our data showing detection of MMP14 in the media is consistent with release of MMP14-TIMP2 complexes in exosomes. However, further experiments are required before we can definitively demonstrate that MMP14 and TIMP2 are components of exosomes released by TM cells.

In summary, we have shown that inducing and inhibiting ECM cross-linking decreased and increased outflow in perfusion culture, respectively. Our results also show that these cross-linking reagents alter MMP levels and activity and affect synthesis of certain ECM proteins. These in turn are likely to change the biomechanics of the tissue and affect tissue stiffness. Our results are consistent with the emerging paradigm that the stiffer the matrix, the lower the aqueous outflow facility through the TM.

## Materials and Methods

### Anterior segment perfusion culture and histology

Human (Lions VisionGift, Portland, OR) and porcine (Carlton Farms, Carlton, OR) anterior segments were perfused at constant pressure (8 mmHg) with serum-free Dulbecco’s Modified Eagles Medium (DMEM) as described previously^50^. The average age of human cadaver eyes was 79.3 ± 2.4 years (range, 61–92). The use of human cadaver tissue was approved by Oregon Health & Science University Institutional Review Board and experiments were conducted in accordance with the tenets of the Declaration of Helsinki for the use of human tissue. BAPN (0.2 mM in serum-free DMEM; Sigma Aldrich, St Louis, MO), genipin (22 μM in dimethyl sulfoxide (DMSO); Sigma Aldrich) or DMSO vehicle control (diluted 1:1000) were applied at time point 0^51^,^52^. Flow rates were measured at least twice a day for a further 70–75 hours. Flow rates after treatment were normalized to the average flow rate before treatment and data from multiple eyes were averaged and a standard error of the mean was calculated^50^. ANOVA was used to determine significance.

Masson’s trichrome stain is a histological stain used to evaluate connective tissues. After removal from the perfusion chambers, the anterior segments were cut into approximately 10 wedges, fixed in 4% paraformaldehyde and embedded into paraffin. Five μm radial sections were cut at the OHSU histopathology core facility (Knight Cancer Institute, Oregon Health & Science University). After deparaffinization and hydration, the sections were stained with Masson’s trichrome stain following the manufacturer’s instructions (Polysciences, Inc., Warrington, PA). This procedure stains collagen blue, nuclei are black and muscle/cytoplasm/keratin is red. Stained sections were viewed on an Olympus BX51 microscope equipped with a DP71 digital camera. At least 3 different eyes were evaluated for each treatment. Representative images are shown.

### Western blotting

Primary human TM cells were prepared and cultured from three different cadaver eyes (average age = 23 ± 11.5 years) using established methods^53^,^54^. TM cells were plated in 6-well plates and grown to confluence. Media was then exchanged to serum-free DMEM containing 0.2 mM BAPN, 22 μM genipin (in DMSO) or vehicle control (DMSO 1:1000). After 48 hours, serum-free media and RIPA lysates were harvested. For cell lysates, protein concentration was determined using the bicinchoninic acid (BCA) assay kit (Pierce Biotechnology, Inc, Rockford, IL) and equal amounts of protein were loaded onto 7.5% SDS-PAGE gels. For serum-free media, proteins in 1 ml of media were precipitated using 20% (w/v) trichloroacetic acid and washed with ice-cold acetone. After separation by 7.5% SDS-PAGE, reduced proteins were transferred to nitrocellulose membranes. Membranes were probed with one or more of the following primary antibodies: MMP14 rabbit polyclonal (ab38971; Abcam, Cambridge, MA), a MMP14 mouse monoclonal (IM57; EMD Millipore, Billerica, MA), MMP2 mouse monoclonal (MAB3308; EMD Millipore), TIMP2 rabbit polyclonal (ab2965; EMD Millipore), collagen type I mouse monoclonal (M-38-c; Developmental Studies Hybridoma Bank, Iowa City, IA), CD44 rat monoclonal (60068; StemCell Technologies, Inc., Vancouver, BC), tenascin C rabbit polyclonal (AB19011; EMD Millipore), fibronectin rabbit polyclonal (ab2413; Abcam), elastin mouse monoclonal (ab9519; Abcam), fibrillin-1 mouse monoclonal (ab9519; EMD Millipore) or versican mouse monoclonal (12C5; Developmental Studies Hybridoma Bank). These primary antibodies were detected with the appropriate species secondary antibody conjugated to either IRDye800 or IRDye700 (Rockland Immunochemicals, Gilbert, PA). The Odyssey IR imaging system (Licor, Lincoln, NE) generated an image of the membrane and pixel density of the bands in each lane was quantitated using ImageJ software. At least three Western immunoblots from HTM cells derived from three different individuals were measured. Data were made a percentage of control and then data from different experiments were averaged and a standard error of the mean was calculated. ANOVA was used to determine significance (p < 0.05).

### MMP and ADAMTS4 activity assays

The SensoLyte 520 generic MMP assay (Anaspec, Inc., Fremont, CA) and a disintegrin and metalloproteinase with thrombospondin-like motifs–4 (ADAMTS4) assay (Sensolyte 520-aggrecanase-1 assay; Anaspec) were performed as described previously^55^,^56^. Briefly, human TM cells were grown to confluence, changed to serum-free media and treated for 24 hours with 0.2 mM BAPN, 22 μM genipin or vehicle control (DMSO diluted 1:1000). Media and cell lysates were collected. For the MMP assay, the MMPs in each media sample were activated with 1 mM 4-aminophenylmercuric acetate for 90 minutes at 37 °C. Samples were incubated with a 5-fluoroscein amidite (FAM)-labeled fluorescent substrate specific for either MMPs or ADAMTS4, which was quenched with QXL520. Following cleavage, the quencher was removed and 5-FAM fluorescence was measured on a plate reader (Ex/Em = 490/520 nm). Relative fluorescence units (RFUs) were then normalized to total protein in each sample, as measured by a BCA assay. Each sample was measured in duplicate and then the data from three biological replicates were averaged, a standard error of the mean was calculated and significance was determined using an ANOVA, where p < 0.05 was considered significant.

### Quantitative RT-PCR

BAPN, genipin and vehicle control-treated cells were harvested using TRIzol (Life Technologies, Carlsbad, CA) and the Direct-zol RNA miniprep kit (Zymo Research, Irvine, CA) was used to purify RNA following the manufacturer’s instructions. The concentration and purity of RNA was then determined using a NanoDrop 2000 (Wilmington, DE). cDNA was generated using 300–600 ng RNA as a template and Superscript III reverse transcriptase (ThermoFisher Sci., Grand Island, NY). For quantitative RT-PCR, the GoTaq qPCR Master Mix (Promega, Madison, WI) was used. Table 1 shows the primer sequences for each of the genes analyzed. A thermal cycler (DNA Engine; Bio-Rad) equipped with a detector (Chromo4; Bio-Rad) was used to amplify products as described previously^41^. All quantitative RT-PCR data were normalized to levels of 18S RNA, which was used as a housekeeping gene. Data were then made a fold change of untreated control cells, averaged and a standard error of the mean was generated. ANOVA was used to calculate whether data were significant (p < 0.05).

## Additional Information

**How to cite this article**: Yang, Y.-F. *et al*. Effects of induction and inhibition of matrix cross-linking on remodeling of the aqueous outflow resistance by ocular trabecular meshwork cells. *Sci. Rep.* **6**, 30505; doi: 10.1038/srep30505 (2016).

## Figures and Tables

**Table 1:** Primers used for quantitative RT-PCR.

Gene	Forward (5′–3′)	Reverse (5′–3′)
COL1A1	CAAAGATGGACTCAACGGTCTC	TGACTGGAAGAGTGGAGAGTAC
CD44	TGCCCAATGCCTTTGATGGA	ATTCTGTCTGTGCTGTCGGT
FN1	TAGTGGAGGCACTGAAAGACCA	AAAGCCTAAGCACTGGCACAAC
FBN1	GAGTGTGCAACCAAGCAACACA	ATGTCTTGGCATCCTCCACTGA
TNC	ATGTGCCCATTACAGGAGGT	AGACAGGCTCGCTTTCCTCAAA
EN	CGCCCAGTTTGGGTTAGTT	AATATGGAGCAGCAGTGCC
VCAN	TGGCACAAATTCCAAGGGCAGT	ATTGCAGTGTGCTGCCATCAGT
18S RNA	CGGCTACCACATCCAAGGAA	CACCAGACTTGCCCTCCAAT
